# Use of palliative radiotherapy in brain and bone metastases (VARA II study)

**DOI:** 10.1186/1748-717X-7-131

**Published:** 2012-08-03

**Authors:** Jose Expósito, Javier Jaén, Enrique Alonso, Isabel Tovar

**Affiliations:** 1 Radiation Oncology Department, Virgen de las Nieves University Hospital, Avd Fuerzas Armadas 4, Granada 18014, Spain; 2 Institute of Oncology Cartuja, Sevilla, Spain; 3 Radiation Oncology Department, Puerta del Mar University Hospital, Cádiz, Spain

**Keywords:** Palliative radiation therapy, Variability, Bone metastases, Brain metastases

## Abstract

**Introduction:**

Metastases are detected in 20% of patients with solid tumours at diagnosis and a further 30% after diagnosis**.** Radiation therapy (RT) has proven effective in bone (BM) and brain (BrM) metastases. The objective of this study was to analyze the variability of RT utilization rates in clinical practice and the accessibility to medical technology in our region.

**Patients and methods:**

We reviewed the clinical records and RT treatment sheets of all patients undergoing RT for BM and/or BrM during 2007 in the 12 public hospitals in an autonomous region of Spain. Data were gathered on hospital type, patient type and RT treatment characteristics. Calculation of the rate of RT use was based on the cancer incidence and the number of RT treatments for BM, BrM and all cancer sites.

**Results:**

Out of the 9319 patients undergoing RT during 2007 for cancer at any site, 1242 (13.3%; inter-hospital range, 26.3%) received RT for BM (n = 744) or BrM (n = 498). These 1242 patients represented 79% of all RT treatments with palliative intent, and the most frequent primary tumours were in lung, breast, prostate or digestive system. No significant difference between BM and BrM groups were observed in: mean age (62 vs. 59 yrs, respectively); gender (approximately 64% male and 36% female in both); performance status (ECOG 0–1 in 70 vs. 71%); or mean distance from hospital (36 vs. 28.6 km) or time from consultation to RT treatment (13 vs. 14.3 days). RT regimens differed among hospitals and between patient groups: 10 × 300 cGy, 5 × 400 cGy and 1x800cGy were applied in 32, 27 and 25%, respectively, of BM patients, whereas 10 × 300cGy was used in 49% of BrM patients.

**Conclusions:**

Palliative RT use in BM and BrM is high and close to the expected rate, unlike the global rate of RT application for all cancers in our setting. Differences in RT schedules among hospitals may reflect variability in clinical practice among the medical teams.

## Introduction

Cancer remains a major health and social problem. Therapeutic advances over the past decade have produced important improvements in cancer control and in the survival of cancer patients, and a better management of their symptoms has enhanced their quality of life [1]. Progress has been made in surgery, chemotherapy and radiation therapy (RT) and in their greater coordination in a multidisciplinary approach [2].

It is estimated that around 20% of patients with solid tumours are diagnosed after the spread of the disease and a further 30% develop metastases at some time after the diagnosis [3]. Treatment for patients with metastases is usually with palliative intent and focuses on the control of symptoms and the maximization of symptom-free time. Metastases are most frequently localized in bone, brain, lung and liver and usually derive from primary tumours in breast, prostate, colon/rectum and lung, i.e., the most frequent solid tumours [1].

As in primary tumours, the best outcomes in metastatic cancer are obtained by adopting a multidisciplinary approach. The use of RT in this setting is supported by considerable and robust evidence. It is considered one of the most effective and cost-effective treatments in patients with bone (BM) or brain (BrM) metastases [4], and palliative RT represents around 10-20% of the total workload in a typical radiotherapy unit [5]. Hypofractionated regimens of short duration are generally prescribed for these patients, although a wide range of regimens and combinations has been applied [6,7]. Comparative data on RT utilization rates across different hospitals are of interest to indicate the access of patients to this technology, its appropriateness and the variability in medical practice [8]. There have been reports of variations in the use of RT for different tumours [9-11] and in the dose schedules selected for BM and BrM among different centres [12-16]. In our region, a previous study found a suboptimal RT utilization rate and significant variability in the use of RT differences among hospitals [17,18]. This finding prompted the present investigation into the use of RT in BM and BrM, two well-established indications for this treatment.

**Figure 1:**
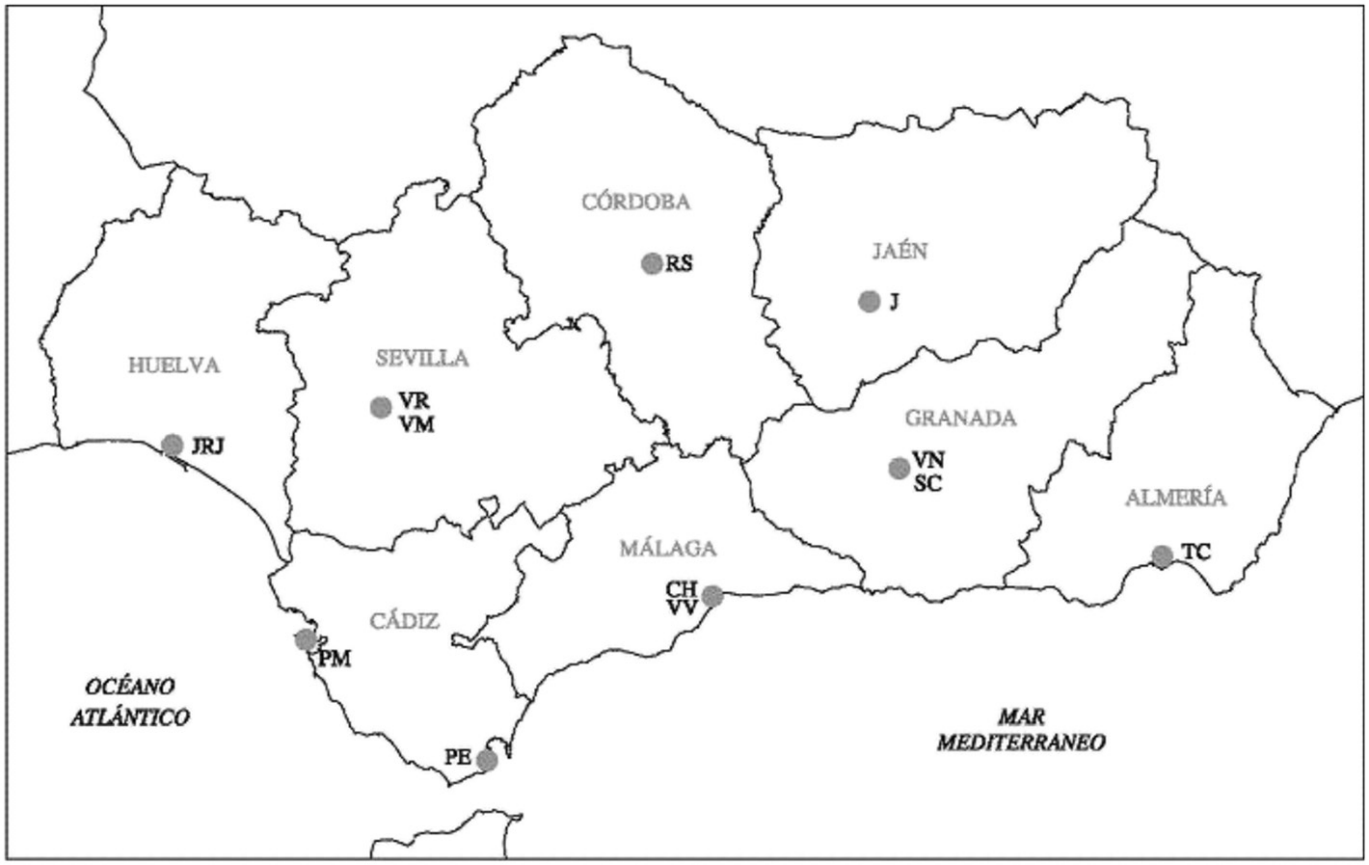
**Location of radiotherapy departments in Andalusia.** TC – Torrecárdenas Hospital (Almería). VN - Virgen de las Nieves University Hospital (Granada). SC - San Cecilio University Hospital (Granada). J - Jaén Hospital (Jaén). RS - Reina Sofía University Hospital (Córdoba). CH - Carlos Haya University Hospital (Málaga). VV - Virgen de la Victoria University Hospital (Málaga). PE - Punta de Europa Hospital (Algeciras, Cádiz). PM - Puerta del Mar University Hospital (Cádiz). VM - Virgen del Rocío University Hospital (Sevilla). VR - Virgen de la Macarena University Hospital (Sevilla). JRJ - Juan Ramón Jiménez Hospital (Huelva).

Andalusia has a surface area of 87597Km^2^ and 7.8 million inhabitants; around half (45.6%) of the population lives within a 20 km radius around the eight cities in the region. The regional public health system provides universal free coverage, while 10% of the total care is delivered in private healthcare facilities. Figure 1 shows the distribution of RT departments in the region.

This study focuses on the RT utilization rate and the patterns of palliative RT application in patients with BM and BrM. It is part of a broader investigation (Variability and Appropriateness of Radiotherapy in Andalusia [VARA] projects I and II) into the quality of RT delivered in 12 public hospitals in the Andalusian public health system [17].

### Patients and methods

This retrospective longitudinal study included all public hospitals in Andalusia (Spain) equipped with RT devices (n = 12, H1-H12); the study period was from January 1 to December 31 2007; data were gathered on all patients treated with external RT for BM or BrM, including details of all palliative RT treatments. Patients were identified from the clinical management computer system linked to the RT equipment (Varis®, lantis® or Impac® Departmental networks) and from the hospitals’ admission records. The same sources were used to gather data on all patients irradiated in any cancer site during the study period. Clinical records and RT sheets were reviewed by specifically trained researchers. When errors or incongruous results were detected, a second review was performed by another member of the research team.

Data were gathered on the type of hospital and RT unit and on the demographic and clinical characteristics and RT treatments of the patients. Study variables included characteristics of the hospital (province), patient (age, gender, type [brain/bone], performance status with Eastern Cooperative Oncology Group [ECOG] scale or Karnofsky scale and primary tumour site), and treatment (medical indication: total doses, fractions, delay after decision, radiosurgery/surgery in brain metastasis, and adverse effects [acute grade 2-3 toxicity]). The distance from RT facilities was based on the area code directory. Because of the difficulty of identifying all patients in the region with indications for palliative RT, we had to estimate the total number. We calculated that palliative RT treatment would be indicated in 1576 patients, based on the cancer incidence of 28144 cases/year [19] and assuming an RT rate (irradiated cases divided by diagnosed cancer cases) of 28%, following VARA I criteria [17,18], and that 20% of RT treatments would be palliative treatments for BM and BrM [20]. We also estimated the percentage of RT candidates per hospital. Toxicity data were retrospectively gathered from clinical records.

#### Statistical procedures

Descriptive outcomes are shown as means, medians, standard deviations and confidence intervals. The chi-square test was used for the comparison of independent qualitative variables.

## Results

During 2007, 1242 patients underwent RT for BM (n = 744) or BrM (n = 498), i.e., 13.4% (inter-hospital range, 5-26.3%) of all patients receiving RT (9310 cases) in the 12 hospitals in the study. These 1242 patients represented 79% of the 1576 patients with BM or BrM estimated to be candidates for palliative RT. The characteristics of the patients are reported in Table 1. Mean age was 62 yrs in BM patients and 59 yrs in BrM patients; the sex distribution was similar in both groups (63% male, 37% female).

**Table 1:** Characteristics of patients

	**Bone Metastasis N = 746**	**Brain Metastasis N = 499**
Age (years)	62 (95%CI: 60–64) Range: 20-92	59 (95%CI: 56–61) Range: 20-79
Gender:		
Male	63%	65%
Female	37%	35%
Primary Site:		
Lung	31%	56%
Breast	26%	20%
Prostate	14%	
Digestive	9%	8%
Other Urologic	6%	3%
Gynaecologic	2%	
Head and Neck	1%	
Others	11%	13%
Location:		
Thoracic spine	34%	
Pelvis	23%	
Lumbar spine	22%	
Femur	9%	
Humerus	3%	
Others	9%	
Radiosurgery:		3.2%
Surgery:		2.4%
Spinal cord compression	10%	
ECOG		
0	24%	31%
1	46%	40%
2	18%	21%
3	9%	5%
4	3%	3%

### Bone metastases (BM)

The primary tumour was lung, breast and prostate in 30.8, 24.6 and 14.3% of BM cases, respectively. In more than two-thirds of cases (79%), BM were in pelvis and vertebra (cervical 7.5%, dorsal 75.5% and lumbar 17%). Patients had varying degrees of medullary compression, while the Eastern Cooperative Oncology Group (ECOG) performance status was 0–1 in 70% of the patients at the start of RT.

Patients lived at a mean distance of 36 Km (95% CI 27–44) from the hospital. The mean delay from radiation unit consultation to RT initiation was 13 days (95% CI 10–15); this delay was 3 days for patients with spinal cord injury, and it was ≤ 7 days in 45% of all BM patients [Tables 2, 3]. RT was an outpatient treatment in 73% of these patients; 53% of treatments were on a Monday or Friday.

**Table 2:** Distance to RT unit and delays

	**Bone Metastasis**	**Brain Metastasis**
Distance (Km)		
Range	0–365	0– 46
Mean (95%CI)	36 (27–44)	28 (22–35)
Median	21	22
Delays (days)		
Range	0– 50	0–67
Mean (95%CI)	13 (10–15)	14 (12–17)
Median	8	10
Delays (%)		
< 7 days	65%	45%
>7 days	35%	55%
In-patients (%)		
No	73%	56%
Yes	27%	44%

**Table 3:** Comparison of hospitals: delay from radiation unit consultation to radiotherapy initiation

**H**	**D BM (TD)**	**D BrM (TD)**
1	8.63 (6.12)	7 (11.68)
2	11.45 (6.92)	7.45 (5.47)
3	9.15 (13.35)	9.90 (13.12)
4	9.16 (11.73)	13 (7.41)
5	13,10 (16.81)	20.36 (10.99)
6	-	-
7	10.21 (11.48)	4.81 (4.19)
8	8.23 (18.10)	4.44 (6.45)
9	6.02 (6.41)	4.93 (4.49)
10	13.15 (20.87)	6.85 (16.58)
11	3.70 (8.83)	5.88 (11.60)
12	2.55 (9.89)	6.60 (20.97)
T	8.27 (12.55)	7.93 (11.91)

H = Hospital.

D BM = mean delay (days) from RT unit consultation to RT initiation for Bone M.

D BrM = mean delay (days) from RT unit consultation to RT initiation for BrM.

TD = typical deviation.

Computed tomography (CT)-based planning was performed before RT in 88% of cases. The megavoltage machine was a Co60 unit in 42.9% of cases. Grade 2–3 toxicity was observed in 13% of patients [Table 4]. A mean of 8% of all RT treatments were for BM, with significant (p < 0.005) inter-hospital differences in this percentage, which ranged from 2.8% in H1 to 16.3% in H9 [Table 5]. The mean distance between residence and hospital was significantly higher in two hospitals (H5 and H8) than in the rest, and significantly longer delays before RT were found in two hospitals (H5 and H10). The mean total RT dose was 22.3 Gy (range 5-44 Gy) and the median was 30 Gy. The regimen was 10 × 300cGy in 32% of cases, 5 × 400 cGy in 27% and 1 × 800 cGy in 25% [Table 4].

**Table 4:** Treatment features

	**Bone M**	**Brain M**
Doses (Gy)		
Range	(5–44)	(18–32)
Mean (95%CI)	22 (21–24)	23 (21–24)
Median	30	20
Dose fraction:		
10 × 300 cGy	32%	58%
5 × 400 cGy	27%	20%
1 × 800 cGy	25%	3%
15 × 200 cGy	2%	19%
3 × 600 cGy	1%	
Others	13%	
CT planning:		
Yes	88%	79%
No	12%	21%
Energy:		
Co60	43%	48%
LA 6 Mv	13%	1%
LA ≥ 15 Mv	44%	51%
Toxicity grade 2-3	13%	15%

**Table 5:** Comparison of hospitals: irradiation rate

**H**	**RTcases**	RT BM(*)	RT BrM(*)	**RT BM + BrM**
1	458	2.8%	2.6%	5.4%
2	128	13.2%	10.9%	24.1%
3	1081	6.8%	6.2%	13%
4	1111	8.5%	3.8%	12.3%
5	827	6.2%	4.2%	10.4%
6	430	8.4%	7.9%	16.2%
7	634	14%	4.7%	23.6%
8	541	4.9%	6%	10.9%
9	698	16.3%	10%	26.3%
10	999	6.45%	4.2%	10.6%
11	1647	7.6%	5.2%	13%
12	765	4.8%	4.2%	5%
T	9319	8%	5.4%	13.4%
				Mean 14.6%

(*) Statistical significance: p < 0.005.

H = Hospital. RT = Radiotherapy. BM = Bone Metastases. BrM = Brain metastases.

RT BM = percentage of cases treated by RT for BM.

RT BrM = percentage of cases treated by RT for BrM.

There were significant (p < 0.0005) inter-hospital differences in the regimens prescribed for these patients [Table 6]: 10 × 300cGy was used by four hospitals (H2, H3, H4, H6 and H12), 5 × 400 by two hospitals (H1 and H10) and 1 × 800cGy by the other three hospitals (H5, H7 and H11).

**Table 6:** RT schemes used in Bone Metastases by hospital*

**H**	**1** × **800cGy**	**5** × **400 cGy**	**10×300cGy**	**Others**	**Total**
1		8	5		13
		61.5%	38.5%		
2		2	14	1	17
		11.8%	82.3%	5.9%	
3	12	8	45	9	74
	16.2%	10.8%	60.8%	12.2%	
4	3	12	63	16	94
	3.2%	12.8%	67%	17%	
5	36	14	2		52
	69.3%	26.9%	3.8%		
6	2	6	24	4	36
	5.6%	16.7%	66.7%	11%	
7	57	20	7	6	90
	63.3%	22.2%	7.8%	6.7%	
8	1	5	1	20	27
	3.7%	18.5%	3.7%	74.1%	
9	16	36	8	54	114
	14%	31.6%	7%	47.4%	
10	9	31	20	4	64
	14.1%	48.4%	31.3%	6.3%	
11	44	46	26	10	126
	34.9%	36.6%	20.6%	7.9%	
12	4	8	25		37
	10.8%	21.6%	67.6%		
T	184	196	240	124	744
	24.7%	26.3%	32.3%	16.7%	

* Statistical significance: p < 0.0005.

### Brain Metastases (BrM)

The primary tumour was in the lung (56%), breast (20%) or digestive system (8%). Performance status was ECOG 0–1 in 71% of BrM patients. These patients lived at a mean distance of 28.5 Km (95% CI 22–35) [Tables 1, 2]. The mean delay from first consultation to RT was 14.3 days (95% CI 11.5-17.19 d), and it was ≤7 days in 45% of cases [Table 3]. RT was an outpatient treatment in 56% of cases. Stereotactic radiosurgery was carried out in 16 patients (3.2%) and surgery in 12 (2.4%).

CT-based planning was performed in 79% of cases. A mean of 5.34% of all RT treatments were for BrM, with significant (p < 0.005) inter-hospital differences in this percentage, which ranged from 2.6% in H1 to 10.9% in H2 [Table 5]. Again, the mean distance between residence and hospital was significantly longer in H5 and H8 and the delay to RT initiation was significantly longer in H5 and H4. There were significant (p < 0.001) interhospital differences in the regimens prescribed for these patients [Table 7], with 10 × 300cGy being used by seven hospitals (H1, H2, H4, H5, H6, H11 and H12) and 3 × 600 cGy by two (H3 and H7).

**Table 7:** RT schemes used in Brain Metastases by hospital*

**H**	**3** × **600cGy**	**5** × **400 cGy**	**10** × **300cGy**	**Others**	**Total**
1		3	8	1	12
		25%	66.7%	8.3%	
2			13	1	14
			92.9%	7.1%	
3	58		9	1	68
	85.3%		13.2%	1.5%	
4	1	2	38	1	42
	2.4%	4.7%	90.5%	2.4%	
5			35		35
			100%		
6			28	6	34
			82%	18%	
7	24	1	5		30
	80%	3.3%	16.7%		
8		22	10	1	33
		66.7%	30.3%	3%	
9	10	53	5	1	69
	14.5%	76.8%	7.3%	1.4%	
10		41		1	42
		97.6%		2.4%	
11		16	63	8	87
		18.4%	72.4%	9.2%	
12			32		32
			100%		
T	93	138	246	21	498
	18.7%	27.7%	49.4%	4.2%	

* Statistical significance: p < 0.001.

The mean total RT dose was 22.9 (range 21–24) and the median was 20 Gy. The schedule was 10 × 300 cGy in 49% of cases and 5 × 400cGy in 28% [Table 4].

The distance from residence to hospital was not associated with the treatment rate or the delay to RT in either group of patients.

## Discussion

Novel therapeutic approaches have improved the survival of cancer patients, including some with metastases from solid tumours, thereby increasing the demand for palliative RT. The effectiveness of external RT has been widely demonstrated [4,20], and it has been estimated that around 50% of patients with newly diagnosed cancer and 10-20% of relapsed patients are suitable candidates for palliative RT [21,22].

Studies on variations in medical practice are valuable to assess the quality of care and clinical practice [23] but few have been published in the field of oncology. They are generally used to compare treatments among geographical areas [24,25] or to survey medical opinions on specific treatment options for hypothetical clinical scenarios [15,26], and both types of study have revealed a substantial variation in cancer care. A high variability in cancer treatment outcomes has also been highlighted in reports from the EUROCARE programme [27].

Limitations of this study include the relatively short time period considered and the sources of information, with some missing data (see Tables), although the fact that data were gathered from a direct review of clinical records and treatment sheets is a study strength. Finally, it was necessary to estimate the number of potential candidates for palliative RT in each hospital, although our estimation was slightly lower than that reported by Nieder et al. [5].

The distribution of clinical variables and tumour sites in the cancer patients in these hospitals was similar to previous international reports [28]. Although the hospitals in this study were all referral centres for RT, the mean distance from the patient’s home to the RT unit was shorter than in other studies [29]. If the results for two of the hospitals (H5 and H10) are excluded, the median delay from first consultation to RT initiation was 13 days, which can be considered acceptable [8,20]. Treatment schedules varied widely among centres [Tables 6,7, consistent with previous reports in different countries [12-16].

The observed treatment rate was slightly lower than the expected rate but was higher than our group found for other cancer sites [9,18], indicating a greater confidence about the use of RT with palliative rather than radical or curative intent. RT with palliative intent represented 14% of all patients undergoing RT, but the hospitals varied widely and significantly in the selection of treatment regimen. The reasons for this variation are not clear and warrant further investigation, although differences in case mix or in the number of patients receiving adjuvant or radical RT may play a role.

According to our findings, the same type of clinical situation is treated with very different doses (total and per fraction) in our region. This is a frequent observation in BM therapy [12,30] and appears to be more related to the clinical care pattern established in RT units rather than to effectiveness or clinical criteria. In BM patients, the frequency of the standard 1x800 cGy scheme, which is supported by well-conducted studies [16,31-33], was strikingly low (25%).

In conclusion, the rate of palliative RT use for BM and BrM in these hospitals was close to published reports, unlike the use of RT for other clinical purposes. Moreover, the delay before RT initiation was relatively low, and the overall rate of RT utilization appears to have been adequate. However, there was a variation in treatment schedules among hospitals that requires further investigation and suggests a need to develop protocols and training programmes to standardize and improve the care of these patients in our setting.

## Abbreviations

RT: Radiotherapy; BM: Bone metastases; BrM: Brain metastases; VARA: Variability and Appropriateness of Radiotherapy in Andalusia; H: Hospital; CT: Computed Tomography.

## Competing interests

The authors declare that they have no competing interests.

## Authors’ contributions

JE: substantial contributions to conception and design of the study, interpretation of data, draft the manuscript, critically revision for important intellectual content, final approval for publication. JJ: substantial contributions to conception and design of the study, acquisition and analysis of data. EA: substantial contributions to conception and design of the study, acquisition and analysis of data. IT: interpretation of data, contribution to draft the manuscript, critically revision for important intellectual content. All authors read and approved the final manuscript.
